# Co-infection of SARS-CoV-2 and DENV case report in Jazan Region Southwest Saudi Arabia

**DOI:** 10.1186/s12879-026-12575-5

**Published:** 2026-01-29

**Authors:** Tareq K. Khawaji, Amro A. Zalah, Emad E. Wasili, Fatima A. Garn, Rasha Y. Hefzi, Ali I. Ageel, Othman M. Shagri, Ahmed M. Ibrahim, Zaki M. Eisa, Ommer M. Dafalla

**Affiliations:** 1 Molecular Biology Department, The Regional Laboratory, Jazan Health Cluster, Jazan, Saudi Arabia; 2 Saudi Public Health Authority, Jazan, Kingdom of Saudi Arabia; 3 Wegaa Center Laboratory, Jazan, Saudi Arabia

**Keywords:** Co-infection, Dengue virus, COVID-19, Jazan Region

## Abstract

Co-infection with Dengue Virus (DENV) and SARS-CoV-2 presents a significant public health challenge, particularly in regions where dengue is endemic. Such cases have been reported in various countries, including Saudi Arabia, particularly in Jeddah. We report the first documented case of dengue and COVID-19 co-infection in the Jazan region of southwestern Saudi Arabia. A 41-year-old female presented with high fever, headache, fatigue, and pansinusitis-like symptoms. During her hospital admission, laboratory investigations revealed thrombocytopenia and severe leukopenia on a complete blood count (CBC) test, symptoms, and blood tests suggestive of dengue fever. RT-PCR confirmed concurrent DENV serotype 2 and COVID-19 infection. The patient was managed symptomatically with intravenous fluids and paracetamol, showing full recovery without complications. This case highlights the need for simultaneous testing and careful differentiation between DENV and SARS-CoV-2 infections in endemic regions.

## Introduction

Co-infection by Corona Virus Disease-2019 (COVID-19) and dengue virus (DENV) can pose a large-scale public health threat, especially in endemic dengue regions with ongoing COVID-19 transmission [1]. Both viruses have different modes of transmission, dengue is transmitted through the bite of an infected female Aedes species mosquito (*Ae. aegypti* or *Ae. albopictus*) [2]. COVID-19 is a respiratory illness, mostly characterized by fever and cough and it spreads via respiratory droplets. COVID-19 was first identified in Wuhan, Hubei, China, in December 2019 [3], has caused more than 5.7 million deaths globally, and infected 396 million people [4]. Co-infection can complicate diagnosing and treating each of these infections because many of the symptoms are similar, including fever, fatigue, body aches and laboratory characteristics (like thrombocytopenia and leukopenia), This overlap can lead to misdiagnosis, particularly in dengue-endemic regions, and may delay appropriate treatment [5, 6].

Dengue virus is the etiological agent of dengue fever, a potentially severe viral illness characterized by high fever, severe headache, pain behind the eyes, joint and muscle pain (muscle and bone pain being so strong that in its earliest entry stemmed one of the “break-bone fever definitions”), and rash. Severe complications of dengue hemorrhagic fever and shock syndrome are potentially lethal [7]. The dengue virus can infect 400 million people with 40,000 deaths in tropical and subtropical regions of the worldwide [8].

Globally, cases of co-infection have been documented in countries like Brazil, Indonesia, Thailand, India, Pakistan, Colombia, Mexico, the Philippines, Malaysia, and Singapore [9–11]. In Saudi Arabia, Khalil et al. [12] and Al-Nazawi et al. [13] have reported co-infection.

The dengue Control Program in the Jazan region, KSA, reported that there were 4192 PCR-confirmed dengue cases between 2005 and 2019 with the highest out-breaks occurring in 2019 (1623 cases), and there are three serotypes (den1, den2 and den3) circulating in the region [14]. The emergence of an imported variant of dengue virus serotype 2 in the Jazan region was reported during the 2019 out-break [15].

To our knowledge, this is the first documented case of COVID-19 and dengue virus co-infection in the Jazan region, southwestern Saudi Arabia, emphasizing the diagnostic complexity caused by overlapping symptoms and hematological abnormalities.

## Case presentation and investigation

A 41-year-old Saudi female patient living in Jazan, was admitted to the Hospital in April 2023 because of chronic pansinusitis symptoms, including headache, fatigue, pain around the eyes, difficulty in breathing, high fever (39 °C), slight complaints of myalgia, nausea, and problems in smelling and testing. No petechiae, rash, or bleeding tendency was observed. Blood pressure remained normal, and oxygen saturation was 98% on room air; thus, no oxygen therapy was required. The initial Complete Blood Count (CBC) and auto-differentiation test showed thrombocytopenia (low platelet count, 90 × 10³/µL), severe leukopenia (low white blood cell count, 1.9 × 10³/µL) and a slight decrease in hemoglobin testing, and whole blood sample was used for dengue virus testing. For the covid19 and dengue fever, automated extraction was performed using the Ez1purification kit (Qiagen Company,-Germany), following the manufacturers protocol for isolating viral RNA.

To detect covid19, a BGI kit (England) was used according to the manufacturer’s protocol. The Altona RealStar^®^ Dengue RT-PCR Kit 2.0 (Germany) was used for dengue virus detection according to the manufacturer’s protocol.

The Covid19 and Dengue RT-PCR tests were positive for the presence of COVID-19 and dengue virus.

Further investigation of dengue virus serotyping using the VIASURE Dengue Serotyping Real-Time PCR Detection Kit (Certest Biotec. Spain) was used, and dengue virus serotype 2 was detected.

The patient was managed with supportive treatment including intravenous fluids, paracetamol, and rest. No antiviral or steroid therapy was required. The condition improved gradually within five days. A follow-up CBC performed one week later showed normalization of platelet (220 × 10³/µL) and white blood cell counts (5.8 × 10³/µL). No progression to dengue hemorrhagic fever or shock syndrome was observed, and the patient fully recovered.

## Discussion

Dengue virus co-infection with the COVID-19 responsible for COVID-19 involves a discussion of symptom overlap, possible interactions, diagnostic difficulties, and public health implications.

Dengue fever and COVID19 share symptoms, such as fever, headache muscle pain, and tiredness making it challenging for doctors to accurately diagnose them clinically. Furthermore, dengue may also present signs, such as cough, which are frequently linked to COVID19. This similarity in symptoms can result in misdiagnosis, and consequently, delayed care. Missed diagnoses of one illness can occur once an other is confirmed, especially in areas where dengue is prevalent [16–18].

Similar to previous reports from Jeddah [12, 13], our patient presented with fever, myalgia, and thrombocytopenia but without hemorrhagic manifestations. Unlike fatal co-infection cases reported in Jeddah and Brazil [12, 13, 16], this patient had a mild, self-limiting course, possibly due to early detection and absence of comorbidities. These differences highlight the variable clinical spectrum of DENV and SARS-CoV-2 co-infection and the importance of close clinical monitoring and laboratory follow-up.

Misdiagnosis becomes more challenging because of constraints in assessments in areas with a shortage of resources. Dengue IgG, which reveals previous encounters with DENV, may mistakenly interact with the antigens of COVID-19, resulting in incorrect positive results. This confusing interaction raises the possibility of wrongly diagnosing one illness over an-other, impeding efforts for treatment and control methods, as stated by Yan et al., 2020 [19].

Co-infection with DENV and COVID-19 may affect the severity of the infection through interactions between the two viruses. Dengue fever alone induces an extremely strong immune response, including cytokines that add to the cytokine storm observed in severe cases of COVID-19. Therefore, co-infected patients are likely to be at a higher risk because the compounding of immune responses might lead to severe disease outcomes, inflammation, vascular permeability, and multi-organ failure [20].

Such interactions between the immune responses can lead to immune dysregulation. In patients with previous DENV infection, antibody-dependent enhancement (ADE) can be a concern; viral entry into host cells facilitated by non-neutralizing antibodies may worsen COVID-19 infection. However, although there have been studies on ADE in DENV infection, this phenomenon in COVID-19 is yet to be investigated [21].

The simultaneous presence of dengue and COVID-19 poses significant public health challenges, especially in tropical and subtropical regions where dengue virus (DENV) is common. In these locations, healthcare systems may face increased pressure from overlapping outbreaks, resulting in resource shortage. To effectively manage these situations, healthcare providers must evaluate both infections in patients who show symptoms of fever and respiratory issues, particularly during peak dengue seasons [22].

Additionally, it is crucial to implement integrated vector control and monitoring strategies, as reducing mosquito-borne transmission can help alleviate the impact of co-infections. Strengthening laboratory capabilities for accurate diagnosis and establishing clear management guidelines for patients with co-infections are vital measures to decrease morbidity and mortality.

In summary, having both DENV and COVID-19 at the time creates a situation, for diagnosing and treating illnesses, as well as managing public health concerns because of similar symptoms and possible immune system effects leading to severe illness. It is crucial to establish procedures and public health measures while delving deeper into the biological processes involved in co infection to reduce the potential dangers associate with this double affliction.

## Limitations

This case report has some limitations. First, serological testing for dengue IgM/IgG and COVID-19 antibodies was not performed, and it may have added valuable insight into the stage of infection and immune interplay. Second, viral load quantification and cytokine profiling were not performed due to limited laboratory resources, and this may have clarified the infective immunopathological mechanism behind co-infection. Finally, there was no long-term follow-up to assess any possible delayed or post- infectious complications.

Taking these limitations into account, this report presents clinical and diagnostic evidence as the first case of documented COVID-19 and dengue virus co-infection in the Jazan region, and emphasizes the need for improved diagnostic capacity and clinician awareness in dengue endemic areas.

## Data Availability

Not applicable.

## Abbreviations

SARS-CoV-2: Severe Acute Respiratory Syndrome Coronavirus 2
COVID-19: Corona Virus Disease – 2019
DENV: Dengue Virus
CBC: Complete Blood Count
RT-PCR: Reverse Transcriptase Polymerase Chain Reaction
ADE: Antibody-dependent enhancement
